# Approaches for estimating minimal clinically important differences in systemic lupus erythematosus

**DOI:** 10.1186/s13075-015-0658-6

**Published:** 2015-06-03

**Authors:** Sharan K Rai, Jinoos Yazdany, Paul R Fortin, J Antonio Aviña-Zubieta

**Affiliations:** Arthritis Research Canada, 5591 No. 3 Road, Richmond, BC V6X 2C7 Canada; Department of Experimental Medicine, University of British Columbia, Vancouver General Hospital, 10226-2775 Laurel Street, Vancouver, BC V5Z 1M9 Canada; Department of Medicine, University of California, 1001 Potrero Ave, San Francisco, CA 94143 USA; Centre de recherche du CHU de Québec, Division de Rhumatologie, Département de Médecine, Université Laval, 2705 boulevard Laurier, Québec, QC G1V 2L9 Canada; Division of Rheumatology, Department of Medicine, University of British Columbia, Suite 802 – 1200 Burrard Street, Vancouver, BC V6Z 2C7 Canada

## Abstract

A minimal clinically important difference (MCID) is an important concept used to determine whether a medical intervention improves perceived outcomes in patients. Prior to the introduction of the concept in 1989, studies focused primarily on statistical significance. As most recent clinical trials in systemic lupus erythematosus (SLE) have failed to show significant effects, determining a clinically relevant threshold for outcome scores (that is, the MCID) of existing instruments may be critical for conducting and interpreting meaningful clinical trials as well as for facilitating the establishment of treatment recommendations for patients. To that effect, methods to determine the MCID can be divided into two well-defined categories: distribution-based and anchor-based approaches. Distribution-based approaches are based on statistical characteristics of the obtained samples. There are various methods within the distribution-based approach, including the standard error of measurement, the standard deviation, the effect size, the minimal detectable change, the reliable change index, and the standardized response mean. Anchor-based approaches compare the change in a patient-reported outcome to a second, external measure of change (that is, one that is more clearly understood, such as a global assessment), which serves as the anchor. Finally, the Delphi technique can be applied as an adjunct to defining a clinically important difference. Despite an abundance of methods reported in the literature, little work in MCID estimation has been done in the context of SLE. As the MCID can help determine the effect of a given therapy on a patient and add meaning to statistical inferences made in clinical research, we believe there ought to be renewed focus on this area. Here, we provide an update on the use of MCIDs in clinical research, review some of the work done in this area in SLE, and propose an agenda for future research.

## Introduction

A minimal clinically important difference (MCID) is an important concept used to determine whether a medical intervention improves perceived outcomes in patients. Prior to the introduction of the concept in 1989, studies focused primarily on statistical significance [1]. As clinicians, investigators, and policy-makers are becoming increasingly interested in incorporating patients’ attitudes, priorities, and perspectives on disease in the longitudinal evaluation of novel intervention strategies, questionnaires assessing health-related quality of life (HR-QOL) and perceived health status are gaining widespread use. However, despite the abundance of such instruments, their interpretability poses a challenge to investigators. MCID directly addresses the limitations of examining statistical significance in isolation, particularly the possibility that studies may find statistical relationships that do not have clinical importance to patients, clinicians, or policy-makers.

Systemic lupus erythematosus (SLE) is a chronic, multi-system autoimmune disease that illustrates some of the challenges posed by defining and measuring MCIDs. SLE is a heterogeneous disease with a wide variety of symptoms in individual patients and across the population. Furthermore, SLE is characterized by periods of low disease activity alternating with periods of higher disease activity, a pattern that directly impacts the patient’s quality of life [2]. However, outcome measures that capture the complexity of SLE and adequately reflect the broad array of symptoms and signs have been challenging to both develop and apply, and limited work has been done to define MCIDs for existing patient-reported measures [3]. Although there has been work applying generic health status instruments in SLE (for example, the Medical Outcomes Study 36-Item Short Form (SF-36), for which an improvement of 2.5 points has been defined as the MCID in SLE [4, 5]), these tools are known to have relatively poor responsiveness in SLE [6, 7]. Thus, there is a need to bring new focus and methodology to MCID measurement in SLE. Here, we provide an update on the use of MCIDs in clinical research, review some of the work done in this area in SLE, and propose an agenda for future research.

## Defining a clinically meaningful difference

The MCID has been proposed as the ‘smallest difference in score in the domain of interest which patients perceive as beneficial and which would mandate, in the absence of troublesome side effects and excessive cost, a change in the patient’s management’ [1]. The MCID therefore constitutes a threshold for outcome scores (either patient-reported or physician-measured) over which a patient or physician would consider a given change in score to be meaningful and worthwhile, which is critical for conducting clinical trials in SLE as well as for facilitating the establishment of treatment recommendations for patients [2, 8].

## Minimal clinically important difference in the context of systemic lupus erythematosus

The Belimumab in Subjects With Systemic Lupus Erythematosus (BLISS)-52 [9] and BLISS-76 [10] trials of belimumab (the first drug approved for SLE in over 50 years) employed the SLE Responder Index (SRI), the first composite measure of SLE disease activity that incorporates criteria from three different validated indices: the Safety of Estrogens in Lupus Erythematosus - National Assessment-SLE Disease Activity Index (SELENA-SLEDAI), a Physician Global Assessment, and the British Isles Lupus Assessment Group (BILAG) instrument [11]. This composite index provides a more comprehensive assessment of SLE disease activity because it uses several instruments simultaneously, thus leveraging the relative advantages and disadvantages of various available indices [11]. In addition to the SRI, the Physical Component Summary (PCS) of the SF-36, a generic instrument for measurement of HR-QOL that has been validated for use in SLE clinical trials, was used to assess change in HR-QOL following treatment with belimumab [9, 10]. Results of the trial showed significant improvement in SF-36 scores across both belimumab groups (1 and 10 mg/kg) at week 52, which correlated with an SRI response (as compared with non-responders) [9]. However, as the SF-36 is not a measure of disease activity, it was not included in the SRI [12].

Unlike the BLISS trials, many other recent trials - for example, the Exploratory Phase II/III SLE Evaluation of Rituximab (EXPLORER) [13], the Lupus Nephritis Assessment with Rituximab (LUNAR) [14], and the abatacept [15] trials - have all reported non-statistically significant results. In the case of rituximab, this is especially disappointing in light of several smaller uncontrolled trials that suggested potential efficacy in SLE [16–19]. Thus, the manner in which a response is defined holds the potential to determine whether a clinical trial is deemed a success or a failure [20]. This has been demonstrated in the context of lupus nephritis, in which Wofsy and colleagues [20] aimed to determine which response criteria are most sensitive to differences among treatment groups.

Thus, as several clinical trials in SLE to date have failed to show significant effects, defining the MCID of existing instruments (as well as composite indices, such as the SRI) may be critical for the conduct of interpretable and meaningful clinical trials in SLE, as it will help determine the effect of a given therapy as well as assist with appropriate design of clinical trials by informing the estimation of effect size, thus facilitating sample size calculation.

## Perspectives on the minimal clinically important difference

The MCID can be defined from the perspective of the patient (or the patient’s proxy, such as a caretaker or partner), health-care professionals, or researchers [21, 22]. For example, the patient may consider a meaningful difference to be one that results in a reduction of symptoms or an improvement in function, thus allowing him or her to perform an essential task or to perform tasks more efficiently (for example, with less pain), but this would not necessarily take into account an intervention’s impact on survival or damage [21]. Conversely, a physician may define a meaningful difference to be a change in treatment or disease prognosis [23]. Additionally, the MCID can be further defined from the perspective of society, which would define a meaningful change as one that allows a patient to return to employment, or of the payers (for example, an insurance company), who would define a meaningful change as one that produces a claim closure [22]. Given the diversity of available perspectives, definitions of the MCID may (and likely will) be discordant.

The MCID may also be defined at either the individual or group level (and will vary accordingly). Inferences made at the group level can inform comparisons between different treatments or decisions regarding public policy; conversely, inferences made at the individual level can inform individual clinical treatment decisions [23]. Furthermore, when considering the magnitude necessary for a change to be considered important, larger changes may be required at the individual level, whereas relatively smaller changes may be interpreted as clinically important when considered at the group level [23, 24].

## Health-related quality of life

The discordance between physician and patient perspectives raises the question of who ought to decide what constitutes a clinically meaningful change. Patients’ perceptions of clinically worthwhile changes are influenced by their health status at baseline as well as their expectations, needs, and goals [21]. Conversely, the clinician’s judgment draws upon previous knowledge and experiences, consideration of things that could be treated, and an understanding of physiologic findings that may not be symptomatic to the patient [21]. Thus, it has been suggested that for measures of physical function and quality of life, responsiveness should be based on the subject’s perception of meaningful change but that for measures of impairment or disease activity, the physician may provide the best judgment [21].

To that effect, various international and multidisciplinary bodies (for example, the Outcome Measures in Rheumatology group) have developed a core set of outcome domains for rheumatic diseases (as had originally been done for rheumatoid arthritis clinical trials) [25, 26]. Specifically, they have recommended that the following constitute a core set of domains for SLE clinical trials: disease activity, HR-QOL, adverse events, and cumulative organ damage [25, 26]. Whether some of these domains should be combined into a composite index (as in the SRI) or evaluated individually warrants further investigation.

The importance of including HR-QOL as a core domain cannot be overstated, as it would incorporate the patient’s perspective of the impact of therapy on various physical, social, and psychological aspects of their health. For example, although a new therapy might show a clinically relevant improvement in disease activity as measured by available indices, this improvement may be contrasted by a clinically meaningful worsening in the patient’s HR-QOL (for example, due to side effects). The converse may also be true: subjective improvements (for example, fatigue, an extremely disabling symptom among patients with SLE) may not be captured by a disease activity index. In this case, capturing clinically meaningful changes in fatigue level could provide great insight into the development and acceptance of new therapeutic agents. Indeed, a trial of the efficacy of abatacept reported that although the study’s primary and secondary endpoints were not met, treatment effects were seen in certain exploratory patient-reported measures, such as in the SF-36, problems with sleep, and fatigue [15].

## Methods to determine the minimal clinically important difference

There are various methods to calculate the MCID and each has relative advantages and disadvantages. An extensive review of available methods was published by Wells and colleagues [27], who classified them into nine different approaches. Another review proposed three distinct categories of approaches for defining the MCID: distribution-based (using statistical descriptions of the population), opinion-based (relying upon experts), and predictive/data-driven (using sequential hypothesis formation and testing) [28]. Overall, regardless of the larger framework employed, methods to determine MCIDs can be divided into two well-defined categories: distribution-based and anchor-based approaches [29]. However, despite this dichotomous classification, distribution-based methods confer the most use when they are applied together with a meaningful external anchor [30].

### Anchor-based methods

Anchor-based approaches compare the change in a patient-reported outcome with a second, external measure of change, which serves as the anchor [29]. Given the large selection of external criteria, this approach can be quite varied [31]. The anchor can be either an objective (for example, medication use or health-care utilization) or subjective (for example, patient self-report of improvement or worsening) measure; however, given the limited availability of acceptable objective assessments, few studies have employed an objective anchor [32, 33]. Instead, anchor-based methods generally rely on the use of a subjective assessment (most commonly a global assessment) [32]. Importantly, these anchor-based methods have the advantage of linking the change in a given score to the patient’s perspective (which is captured by the anchor) [23].

According to a comprehensive review by Copay and colleagues [32], four variations of the anchor-based approach can be described: (a) the ‘within-patients’ score change, (b) the ‘between-patients’ score change, (c) the sensitivity- and specificity-based approach, and (d) the social comparison approach. Another extensive review, by Crosby and colleagues [23], summarized various anchor-based methods for determining individual change according to cross-sectional versus longitudinal methods, with longitudinal methods being more linked with change and thus conferring a benefit over cross-sectional methods. To that effect, employing a longitudinal approach is of particular benefit in SLE, which is characterized by fluctuating status due to flares and remissions.

In addition to determining the responsiveness of the SLE Activity Questionnaire (SLAQ) by using the standardized response mean (SRM), the aforementioned study by Yazdany and colleagues [34] further examined the responsiveness of the SLAQ among a large observational cohort of SLE patients by employing clinically relevant and validated patient assessments of disease activity and health status (for example, the SF-36 Physical Functioning subscale) as the anchors. SLAQ scores were found to correlate strongly with these other health instruments (that is, the anchors), with the exception of the Short-Form 12 PCS [34].

### Limitations of the anchor-based methods

First, the application of different anchors or anchor types may produce different values of the MCID [32], although this is not unlike the distribution-based methods in which different statistical approaches will also produce a variety of MCID values. Additional limitations include a potential discordance of defined MCID values based on whether data collection of the anchor was prospective versus retrospective [35], the possibility that the MCID as determined by anchor-based methods falls within the instrument’s random variation [23], and the susceptibility of some ratings to recall bias [23] (although perhaps this may be alleviated by considering the necessity or wish for change in medication at a given time point, rather than the change over time, which warrants further research).

### Distribution-based methods

Distribution-based interpretations are based on statistical characteristics of the obtained samples [29]. There are various methods within the distribution-based approach, including the standard error of measurement (SEM), the standard deviation, the effect size, the minimal detectable change, the reliable change index (RCI), and the SRM.

#### Standard error of measurement

The SEM is defined as the variation in patient-reported outcome scores attributed to instrument unreliability, in which a change smaller than the calculated SEM is likely due to measurement error rather than a true change [32]. Thus, the SEM is considered to be a characteristic of the measure, not the sample [36]. To define the MCID, threshold values of 1 SEM, 1.96 SEM, and 2.77 SEM have been suggested [36–38]. To illustrate, the MCID can be defined by using the SEM of changes in disease activity scores of SLE patients who have stable disease (that is, patients rated as having no change in disease between consecutive visits) [39]. This was done in a recent study that determined the MCID of validated measures of SLE disease activity in childhood-onset SLE [39]. The MCID was based on both the 1-SEM criterion (which makes the assumption that meaningful improvement or worsening has occurred if the change is plus or minus 1 SEM, respectively) and a more strict criterion (that is, ± 1.645 SEM) [39]. A tighter confidence interval resulted in a more accurate detection of patients with stable disease versus those who experienced clinically important change [39].

#### Standard deviation

Another measure of variability is the standard deviation [32], defined as the variation among a group of scores, for which 0.5 standard deviations has been suggested to correspond to the MCID in a number of studies [40]. An example of this method is provided in a study by Katz and colleagues [41], in which changes in valued life activity scores from baseline to the end of the follow-up were defined as clinically meaningful on the basis of the criterion of at least 0.5 standard deviations.

#### Effect size

The effect size is a standardized measure of change obtained by dividing the difference in scores from baseline to post-treatment by the standard deviation of baseline scores [32]. For interpreting effect sizes, Cohen [42] has proposed the following benchmarks: 0.20, 0.50, and 0.80, indicating small, moderate, and large effects, respectively. Practically speaking, the effect size should be small in patients reporting no change and large in patients reporting a great improvement [43]. In a study by Fortin and colleagues [44], the effect size was employed to determine the responsiveness of two lupus activity measures: the revised Systemic Lupus Activity Measure (SLAM-R) and the SLEDAI. Based on this methodology, the SLAM-R performed better than or the same as the SLEDAI for both clinical improvement and worsening. With regard to no change, the two measures performed equally well.

#### Minimal detectable change

A measure of variability associated with the SEM is the minimal detectable change (MDC), which is the smallest detectable change that can be considered above the measurement error with a given level of confidence (usually 95 % confidence) [32]. Although this method has not been used in SLE research to date, we provide an example from a study of a comprehensive rehabilitation intervention among patients with osteoarthritis by using the Western Ontario and McMaster Universities Arthritis Index (WOMAC) and the SF-36 to estimate the MDC and the MCID for improvement [45]. In the WOMAC sections, the MDC ranged from 0.75 (global) to 0.96 (stiffness), whereas in the SF-36 the MDC ranged from 2.8 (PCS) to 7.6 (physical function) [45]. The corresponding values for MCID in the WOMAC sections ranged from 0.51 to 1.33 points (on a scale of 0 to 10) and in the SF-36 the values ranged from 2.0 to 7.8 points (on a scale of 0 to 100), respectively [45]. These findings have implications for the design of meaningful clinical trials, as sections that showed moderate responsiveness (for example, the SF-36 bodily pain) require a relatively lower sample size as compared with sections that demonstrated lower responsiveness (for example, the SF-36 physical function), which require larger sample sizes.

#### Reliable change index

The RCI is a statistic that assesses the magnitude of change necessary for a given self-report measure to be considered statistically reliable. It is calculated by dividing the individual patient change score by the square root of the SEM [32]. The RCI is considered to confer a true change when it is more than 1.96 (95 % confidence) (that is, the *z*-score corresponding to the desired level of significance) [32]. Although we are not aware of the application of this method in SLE research to date, it has been used to determine the clinical significance of the SF-36 [46]. Specifically, the RCIs were calculated to be 7.47 and 9.70 (reported in T-score units, the standard metric for scoring and interpreting the SF-36), corresponding to the PCS and Mental Component Summary, respectively (calculated at the 0.05 level of significance).

#### Standardized response mean

The SRM is similar to the effect size, except the change in score is divided by the standard deviation of that change [23]. Similar benchmarks have been proposed to guide the interpretation of the SRM [47–49]. In the aforementioned study by Fortin and colleagues [44], the SRM was also employed to determine the responsiveness of both the SLAM-R and the SLEDAI. The same pattern was noted for the comparison of the two measures as was reported for the effect size approach [44]. Furthermore, a study by Yazdany and colleagues [34] ascertained the responsiveness of the SLAQ by calculating the SRM and found that the SLAQ demonstrated a small to moderate degree of responsiveness for patients reporting a perceived change in disease status. Although the overall SRM was found to be 0.12 (that is, minimally responsive [42]), after stratification by patient changes in the patient global assessment of disease activity the SRMs were found to be 0.66 and −0.37, corresponding to clinical deterioration and improvement, respectively. Furthermore, the SRM of no change was found to be 0.10. These values are similar to those obtained for other commonly employed disease activity indices among patients with SLE [50].

### Limitations of the distribution-based methods

First, it must be noted that the application of the various distribution-based approaches described above will result in different definitions of the MCID, which contradicts the intended aim of defining a specific threshold [32]. Most importantly, distribution-based methods are limited by their ability to define only a minimal value below which a change in outcome score for a given measure may be due to measurement error [33], which does not provide information on clinical importance. Thus, these methods largely ignore the core of the MCID, which is to define the clinical importance of a given change in outcome scores separate from their statistical significance [32].

### Delphi method

The Delphi technique is a well-used method (opinion-based) for the development of a formal consensus [27, 51] and can serve as a useful adjunct to finalize MCID values following application of either the distribution- or anchor-based methods. The Delphi method involves the presentation of a questionnaire or interview to a panel of individuals in a specific field for the purpose of obtaining a consensus [52]. Participants are initially sent a questionnaire and asked to record their views; then, participants revise these responses after viewing the responses of co-participants, typically by using a Likert scale [51]. These responses are collected by the organizers and re-distributed to participating individuals as a summary of the group’s judgment, as are the individuals’ responses [51]. Despite substantial divergence of individual opinions in the first round of a Delphi investigation, there is a tendency for convergence of opinions toward a consensus after several iterations of this multi-step process (often repeated several times) [52]. This method has been increasingly used to develop classifications as well as response criteria for rheumatic diseases.

Recently, Brunner and colleagues [53] applied the Delphi survey method to achieve consensus on a definition of global flares in juvenile SLE and to derive candidate criteria to measure juvenile SLE flares. Outside the context of SLE, a study determining the MCID in activity limitation, fatigue, and sleep quality among rheumatoid arthritis patients initially used an internal-anchor approach, and after the preliminary MCID values were determined, the Delphi exercise was applied to reach a consensus on the final MCID values [54].

## Recommendations and future research agenda

As most clinical trials in SLE to date have failed to show significant effects, determining the MCID of the instruments used to measure response may be critical for the conduct and interpretability of meaningful future clinical trials. However, little work in MCID estimation has been done in the context of SLE. Given that the MCID can help determine the effect of a given therapy on a patient and add meaning to statistical inferences made in clinical research, we believe there ought to be renewed focus on this area.

Specifically, we propose the following research agenda in the context of MCID.

1. Explicitly involving patients in defining MCIDs. Assessment of subjective yet equally important and disabling disease characteristics (for example, fatigue and physical functioning) holds the potential to incorporate the patient’s perspective in a standardized way, thus facilitating the development of new therapies while making significant contributions that are valued by those who have the disease.
2. Evaluating the patient with homogeneous levels of disease to increase responsiveness. Given the heterogeneity of SLE, we suspect that research will proceed more efficiently if some work attempts to evaluate patients with similar types of SLE (for example, individuals with a flare of lupus nephritis or with similar disease activity - either by organ involvement or global disease - level at baseline).
3. Incorporating health assessment instruments in the MCID as part of the overall assessment of response. General health assessment questionnaires, such as those that evaluate HR-QOL or use state-of-the-art methods to define a broad array of relevant symptoms (for example, the Patient Reported Outcome Measurement System, also known as PROMIS, an accessible ‘item bank’ to measure health concepts applicable to a variety of chronic conditions [55]) will likely be a fruitful area for future research in MCID among patients with SLE.
4. Assessment of individual organ involvement independent from overall disease activity. In addition to the assessment of overall disease activity, of particular importance is the assessment of disease outcome in terms of overall organ involvement, as SLE represents a systemic disease in which disease activity can improve in some organ systems while it worsens in others [8]. This concept is captured in the BILAG index but would benefit from adding the MCID (either alone or as part of the SRI) to further improve responsiveness, although the BILAG may have to be reweighted accordingly.
5. Development of a grading response using the MCID. Research is needed into the potential application of grading response (for example, small, moderate, or large response) in SLE using the MCID (rather than a binary concept), which may be an important discriminatory parameter to measure responsiveness in SLE.
6. Scoring using multiple instruments simultaneously. Finally, to appropriately account for these various domains in a disease as heterogeneous as SLE, it is imperative that we continue to develop a way to score multiple instruments simultaneously. To that end, further research is warranted to better understand the MCID of the various instruments available.
7. Determine whether the MCID is dependent upon the direction of the change in score (that is, clinical improvement versus worsening). As the MCID of a given self-report measure may vary depending on whether the reported change in score is positive or negative, this ought to be considered when scoring these instruments [56].

In summary, as new therapies are urgently required to treat this devastating and debilitating disease, we cannot afford to wait another 50 years before adding another therapy to the armamentarium for SLE management. Thus, to facilitate further development of new therapeutic agents for SLE, it is critical that funding agencies, researchers, patient organizations, and industry sponsors work collaboratively to close existing knowledge gaps on appropriate measurement of response in SLE.

## Abbreviations

BILAG: British Isles Lupus Assessment Group
BLISS: Belimumab in Subjects With Systemic Lupus Erythematosus
HR-QOL: health-related quality of life
MCID: minimal clinically important difference
MDC: minimal detectable change
PCS: Physical Component Summary
RCI: reliable change index
SEM: standard error of measurement
SF-36: Medical Outcomes Study 36-Item Short Form
SLAM-R: revised Systemic Lupus Activity Measure
SLAQ: Systemic Lupus Erythematosus Activity Questionnaire
SLE: systemic lupus erythematosus
SLEDAI: Systemic Lupus Erythematosus Disease Activity Index
SRI: Systemic Lupus Erythematosus Responder Index
SRM: standardized response mean
WOMAC: Western Ontario and McMaster Universities Arthritis Index
